# The crystal structure of *Grindelia robusta* 7,13-copalyl diphosphate synthase reveals active site features controlling catalytic specificity

**DOI:** 10.1016/j.jbc.2024.107921

**Published:** 2024-10-23

**Authors:** Anna E. Cowie, Jose H. Pereira, Andy DeGiovanni, Ryan P. McAndrew, Malathy Palayam, Jedidiah O. Peek, Andrew J. Muchlinski, Yasuo Yoshikuni, Nitzan Shabek, Paul D. Adams, Philipp Zerbe

**Affiliations:** 1 Department of Plant Biology, University of California-Davis, Davis, California, USA; 2 Technology Division, Joint BioEnergy Institute, Emeryville, California, USA; 3 Molecular Biophysics and Integrated Bioimaging, Lawrence Berkeley National Laboratory, Berkeley, California, USA; 4 US DOE Joint Genome Institute, Lawrence Berkeley National Laboratory, Berkeley, California, USA; 5 Department of Bioengineering, University of California Berkeley, Berkeley, California, USA

**Keywords:** protein crystallization, natural product biosynthesis, plant biochemistry, terpenoid, enzyme mechanism, diterpene synthase, plant specialized metabolism

## Abstract

Diterpenoid natural products serve critical functions in plant development and ecological adaptation and many diterpenoids have economic value as bioproducts. The family of class II diterpene synthases catalyzes the committed reactions in diterpenoid biosynthesis, converting a common geranylgeranyl diphosphate precursor into different bicyclic prenyl diphosphate scaffolds. Enzymatic rearrangement and modification of these precursors generate the diversity of bioactive diterpenoids. We report the crystal structure of *Grindelia robusta* 7,13-copalyl diphosphate synthase, GrTPS2, at 2.1 Å of resolution. GrTPS2 catalyzes the committed reaction in the biosynthesis of grindelic acid, which represents the signature metabolite in species of gumweed (*Grindelia* spp., Asteraceae). Grindelic acid has been explored as a potential source for drug leads and biofuel production. The GrTPS2 crystal structure adopts the conserved three-domain fold of class II diterpene synthases featuring a functional active site in the γ*β*-domain and a vestigial ɑ-domain. Substrate docking into the active site of the GrTPS2 apo protein structure predicted catalytic amino acids. Biochemical characterization of protein variants identified residues with impact on enzyme activity and catalytic specificity. Specifically, mutagenesis of Y457 provided mechanistic insight into the position-specific deprotonation of the intermediary carbocation to form the characteristic 7,13 double bond of 7,13-copalyl diphosphate.

Plant labdane diterpenoids form a diverse group of more than 7000 metabolites that have critical biological functions as conserved gibberellin phytohormones controlling developmental processes and as often species-specific specialized metabolites that mediate defensive and cooperative interactions of plants with animals, microbes, and the environment (1, 2, 3). Moreover, diterpenoids have versatile human uses as pharmaceuticals, biofuels, food additives, fragrances, and various other commodity chemicals (4, 5, 6). Among these specialized diterpenoids, grindelic acid and derivatives thereof have potential medicinal uses in cancer treatment and as NADPH oxidase (Nox) inhibitors in the treatment of Alzheimer’s disease (7). Grindelic acid is the signature metabolite of species of the genus *Grindelia* (Asteraceae), which forms the major component of a resinous terpenoid blend that is formed in and secreted from above-ground tissues (8, 9, 10, 11). Due to the high content of grindelic acid, making up more than half of the resin metabolite profile, and the climate tolerance of members of the genus *Grindelia*, grindelic acid and related resin metabolites also have been explored for biofuel production (12).

The first committed reaction in grindelic acid biosynthesis is the transformation of the core diterpenoid precursor geranylgeranyl diphosphate (GGPP) into the bicyclic intermediate 7,13-copalyl diphosphate (7,13-CPP) catalyzed by the class II diterpene synthase (diTPS) *Grindelia robusta* TPS2 (GrTPS2) (9) (Fig. 1). GrTPS2 is mechanistically closely related to other class II diTPS enzymes that facilitate the protonation-dependent cyclization of GGPP to form a range of CPP intermediates via a common labda-13-en-8-yl^+^ diphosphate intermediate (3, 13). Product specificity of class II diTPSs is then determined by different downstream rearrangements and terminal neutralization of the intermediary carbocation, which most commonly include terminal deprotonation at C-17 to yield CPP products of normal (+), *ent*- or *syn*-stereochemistry. Alternate deprotonation at the endocyclic C-7 methylene will result in the formation of 7,13-CPP, while deprotonation at C-9 yields 8,13-CPP (13, 14, 15). Formation of 7,13-CPP through rearrangement and termination of the carbocation at the endocyclic C-7 methylene is an unusual reaction that, outside of the genus *Grindelia*, has only been observed in the Lamiaceae species, *Hyptis suaveolens*, and a bifunctional class II/I diTPS of the lycophyte *Selaginella moellendorffii* that forms the labdane alcohol, labda-7,13*E*-dien-15-ol via 7,13-CPP as an intermediate (14, 16).

**Figure 1:**
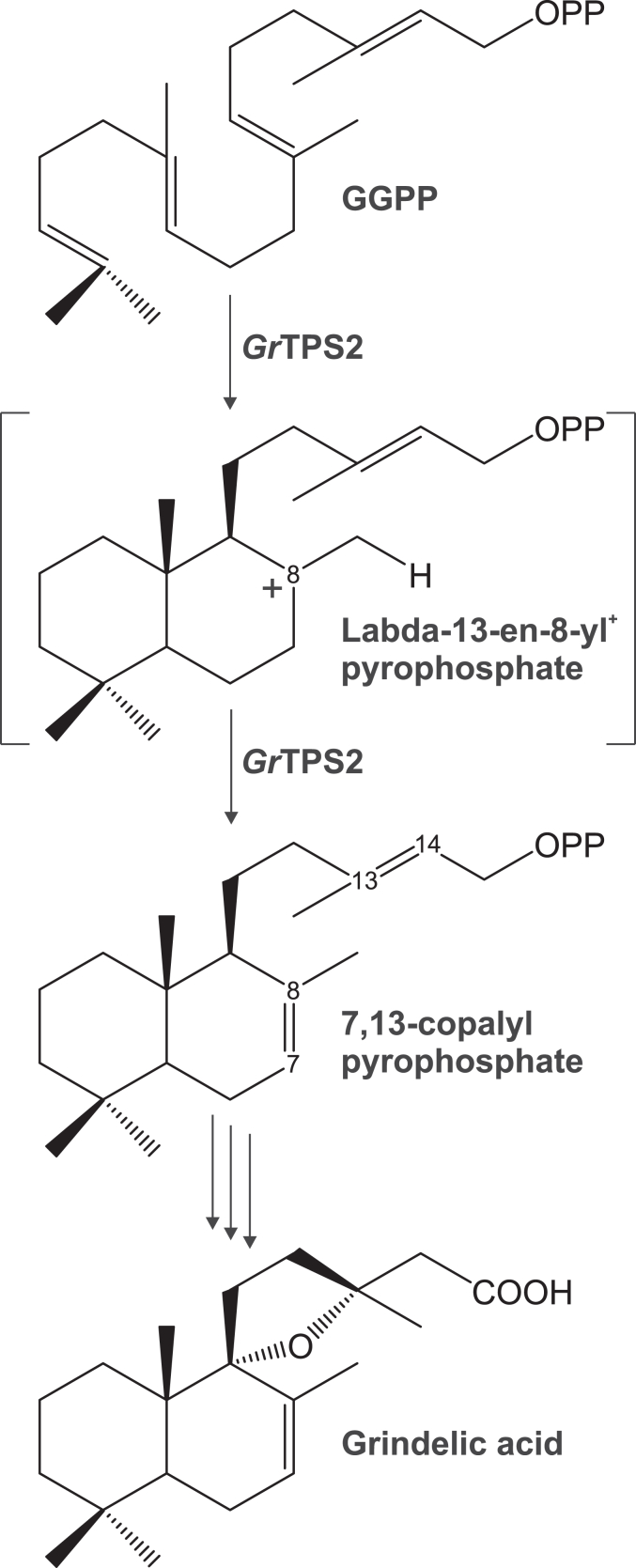
**Schematic overview of the biosynthesis of grindelic acid via 7,13-copalyl diphosphate (7,13-CPP) in *Grindelia robusta*.** The monofunctional class II diterpene synthase, GrTPS2, catalyzes the conversion of the universal diterpenoid precursor geranylgeranyl diphosphate (GGPP) into 7,13-CPP via labda-13-en-8-yl^+^ diphosphate intermediate. Removal of the diphosphate moiety and sequential oxygenation are predicted to occur to transform 7,13-CPP into grindelic acid.

Structural insight into the mechanisms underlying class II diTPS catalysis remains limited with presently only two crystal structures of class II diTPS active sites available, the *Arabidopsis ent*-CPP synthase (AtCPS) and the class II/I enzyme *Abies grandis* abietadiene synthase (17, 18, 19). All class II diTPS share a common three-domain (γβα) fold with the active site being formed by the N-terminal γ and β domains (20, 21). A conserved catalytic DxDD motif controls the general acid-base mechanism to bring about the cyclization of GGPP with the middle Asp residue functioning as the general acid (22, 23). A high-resolution crystal structure of AtCPS further showed that GGPP protonation through this conserved Asp involves proton transfer via a hydrogen-bonded proton wire linking the active site cavity to the bulk solvent (19). Additionally, the functional catalytic base of AtCPS was identified as a water molecule coordinated by a dyad of two His and Arg residues conserved among *ent*-CPP synthases (24, 25). Numerous structure-guided mutagenesis studies further identified active site residues relevant for the terminal deprotonation of the labda-13-en-8-yl^+^ intermediate rather than quenching of the carbocation by hydroxylation at C-8 or C-9 (25, 26, 27, 28), as well as the cascade of possible 1,2-hydride and methyl shifts toward different CPP double bond isomers and enantiomers (15, 29, 30, 31, 32, 33).

This study reports the crystal structure of GrTPS2 at 2.1 Å of resolution. Structural analysis and site-directed mutagenesis studies highlight active site residues contributing to GrTPS2 activity and product specificity toward forming the 7,13-CPP isomer, thus providing insight into the functional divergence of the class II diTPS family.

## Results

**Figure 2:**
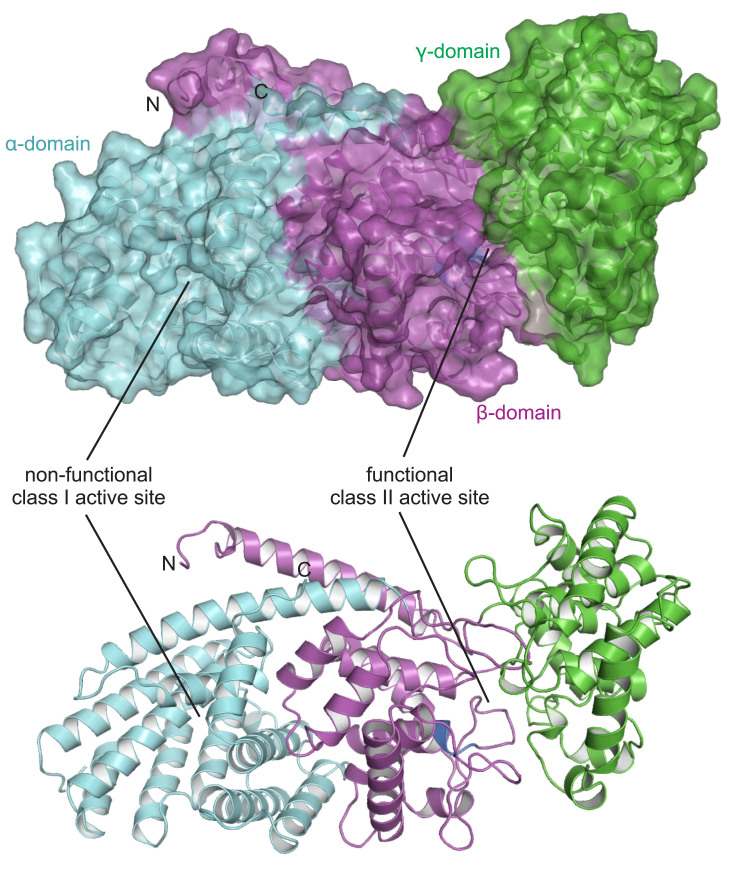
**GrTPS2 structure.** Surface (*top*) and cartoon (*bottom*) representation of the crystal structure of GrTPS2 (PDB ID 9B99) adopting the typical three-domain architecture of class II diterpene synthases, comprising the N-terminal γ-domain (*green*) and β-domain (*magenta*) that harbor the class II active site including the catalytic DIDD motif (*blue*) and the α-domain (*teal*) representing a vestigial class I active site. The N-terminal segment including the first helix forms part of the β- and α-domain.

**Figure 3:**
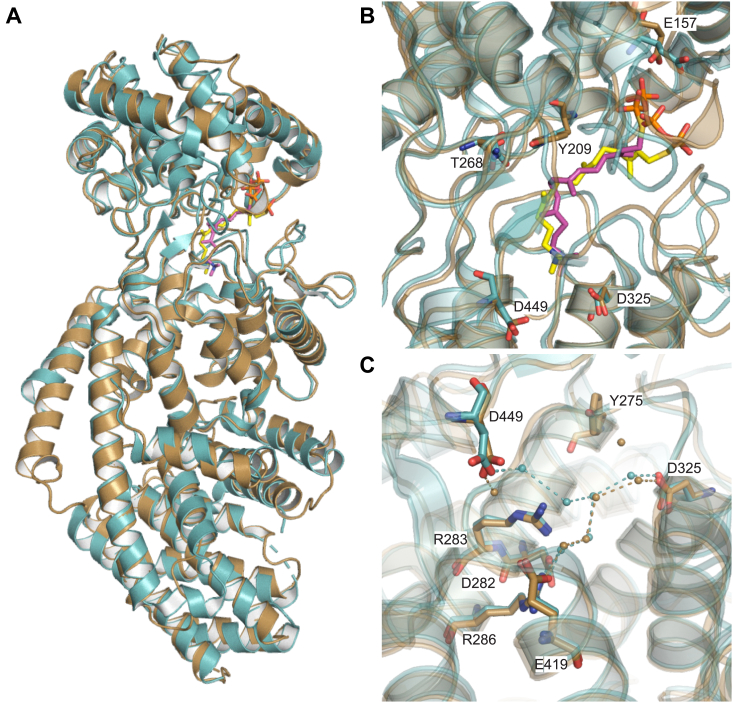
**Structural comparison of GrTPS2 and *Arabidopsis thaliana ent*-copalyl diphosphate synthase.***A*, superimposition of the crystal structure of GrTPS2 (PDB ID 9B99, *brown*) and the crystal structure of *Arabidopsis thaliana ent*-copalyl diphosphate synthase (AtCPS; PDB ID 4LIX; *teal*). The substrate analog, aza-GGSPP (15-aza-14,15-dihydro-geranylgeranyl thiolodiphosphate), present in the AtCPS structure (*yellow*) and docked into the active site of GrTSP2 (*magenta*) are depicted as stick models. *B*, GrTPS2 contains a conserved Glu residue (E157) likely involved in ligand binding as based on prior structural studies of AtCPS. By contrast, Residues of a His-Asn dyad critical for *ent*-CPS product specificity are represented by a Tyr (Y209) and a Thr (T268) in GrTPS2. Aza-GGSPP was docked into the active of GrTPS2 and is depicted as a magenta stick model. *C*, comparison of the GrTPS2 and AtCPS active site showing solvent channels that connect bulk solvent to the general acid of the DIDD motif critical for AtCPS activity. Conservation of relevant active site residues and select water molecules suggest the presence of a similar solvent channel in GrTPS2.

**Figure 4:**
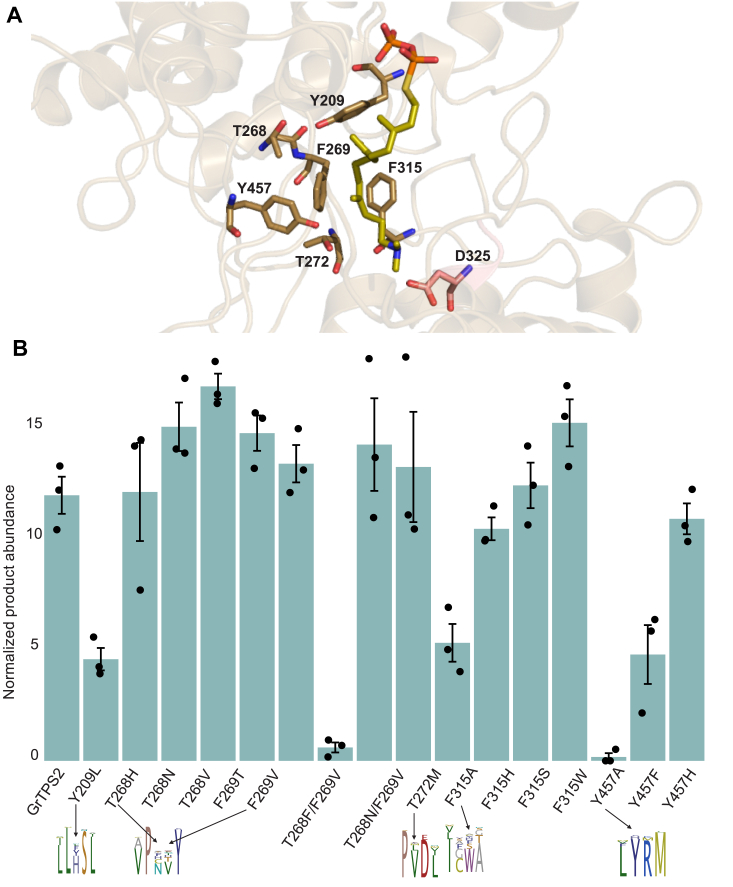
**Structure-function analysis of GrTPS2 protein variants.***A*, crystal structure of GrTSP2 with the substrate analog aza-GGSPP (*dark yellow*; 15-aza-14,15-dihydro-geranylgeranyl thiolodiphosphate) docked into the class II active site located in the γβ-domain. Active site residues proximal to the docked ligand and selected for site-directed mutagenesis in this study are highlighted. *B*, relative abundance of extracted 7,13-copalol (*i.e.* dephosphorylated 7,13-CPP). resulting from *Escherichia coli* co-expression of GrTSP2 and the generated protein variants with *Abies grandis* GGPP synthase. Products were measured via GC-MS analysis and normalized against 1-eicosene as an internal standard. Standard errors are based on three replicates. Residue variation at the individual position across known plant class II diTPSs is highlighted. Error bars represent propagated SE values (n = 3).

The X-ray data collection and statistical analysis of the final refinement of GrTPS2 structure are shown on Table 1. GrTPS2 adopts the common class II diTPS γβα-domain structure comprised of three helical domains that closely resemble the reported structures of the class II *ent*-CPP synthase from *Arabidopsis thaliana* (PDB ID 4LIX), the class I diTPSs *Taxus brevifolia* taxadiene synthase (PDB ID 3P5P), and the bifunctional class I/II abietadiene synthase from *A. grandis* (PDB ID 3S9V) (17, 18, 19, 34). The N-terminal segment (residues 1–18) and a short region of the J-K loop in the α-domain (residues 563–566) are disordered in the GrTPS2 structure (Fig. 2). Consistent with other class II diTPSs, the C-terminal α-domain lacks helices α1 and G1 and helices D, F, H, and I are shorter as compared to class I diTPSs resulting in a non-functional class I active site (Fig. S1). The N-terminal region, including the first helix, folds back to represent part of the β-domain as previously observed also for AtCPS. The GrTPS2 class II active site is formed by the γ- and β-domains, which adopt the typical ɑ-barrel structure and closely resemble the AtCPS active site (Fig. 3*A*). AtCPS E211, predicted to be involved in substrate binding and the catalytic DIDD motif, including D325 as the general acid functioning in substrate protonation (18, 19, 23), is conserved in GrTPS2 (E157) and situated at the bottom of the active site cavity (Fig. 3*B*). By contrast, a His-Asn dyad, previously shown to be important for catalytic specificity in AtCPS and other *ent*-CPP synthases, is replaced with Tyr (Y209) and Thr (T268) residues located at the top of the active site (Fig. 3*B*). Structural analysis of AtCPS had further demonstrated solvent channels, comprised of select active site residues (*e.g.*, D503, R340, R337, D336), and coordinated water molecules that connect the bulk solvent with the general acid Asp residue of the DIDD motif (D379) to facilitate proton transfer during the catalytic reaction (19). The respective GrTPS2 residues, namely D449, R286, R283, and D282, are conserved in GrTPS2 and coordinate several water molecules. Specifically, the general acid D325 forms a water channel to E419 with water molecules W21, W120, W145, and W162 in near-identical position as compared to the solvent channel identified in AtCPS. Additional waters W89, W104, and W165 connect E419 to the D449 residues creating a conserved water solvent network also observed in AtCPS (Fig. 3*C*). Furthermore, another water molecule (W318) is connected to this channel through a hydrogen bond with Y275. To identify additional active site residues with potential impact on GrTPS2 catalysis, the GGPP substrate analog, (*S*)-15-aza-14,15-dihydro-geranylgeranyl thiolodiphosphate (aza-GGSPP), was docked into the GrTPS2 class II active site using a predicted active site cavity and the position of aza-GGSPP in the AtCPS structure (19) as a spatial constraint. All residues present within a 6Å sphere around the docked ligand were selected and further evaluated using protein sequence alignment of GrTPS2 and functionally characterized plant class II diTPSs to identify amino acids either uniquely occurring in GrTSP2 or matching active site positions shown to impact catalytic specificity in other class II diTPSs (Fig. S2). This included Y209 and T268 in the position of the HN dyad critical for product specificity in *ent*-CPP synthases, as well as the neighboring F269 and T272 that, to our knowledge, are unique to GrTPS2. While the aromatic ring of F269 faces the docked ligand with a distance as close as 3.6Å, T272 is situated at the bottom of the active site cleft on the opposite site of the ligand as compared to the general acid DID^325^D motif (Fig. 4*A*). In addition, F315 is positioned in close proximity to the substrate analog, whereas residue Y457 is located 3.5Å away from the ligand. Lastly, Y457 is located in an active site position previously reported to impact product specificity in the rice *syn*-CPP synthase, OsCPS4 (29, 35), the switchgrass class II diTPSs, PvCPS38 and PvCPS3, which produce *syn*-CPP and 8,13-CPP, respectively (15) (Fig. 4*A*).

**Table 1:** Statistics for data collection and refinement of GrTPS2

Dataset	GrTPS2 (PDB ID 9B99)
Data collection	
Wavelength (Å)	0.99999
Resolution range (Å)	58.73–2.12 (2.17–2.12)
Detector Distance (mm)	280
Φ (deg.) collected/ΔΦ (deg.)	180/1.00
Exposure time (seconds)	3
Temperature of collect (Kelvin)	100
Data statistics	
Space group	P 21
Unit-Cell parameters	56.07 79.11 88.44 90 97.59 90
Unique reflections	43361 (2706)
Multiplicity	3.7 (3.7)
Data completeness (%)	99.44 (99.56)
<I/σ(I)>	10.2 (1.4)
(R_merge %_)	0.099 (1.027)
R_pim_ (%)	0.060 (0.618)
CC_1/2_	0.997 (0.566)
Structure Refinement	
Reflections used in refinement	43361 (2706)
Reflections used for R_free_	2178 (142)
R_factor_	0.185 (0.283)
R_free_	0.228 (0.331)
RMS from ideal geometry	
Bond lengths (Å)	0.003
Bond angles (º−)	0.55
Average B-factor	47.5
Macromolecules	47.7
Solvent	43.1
Ramachandran Plot	
FavoreQd region (%)	96.9
Outliers region (%)	0.28
Rotamer outliers (%)	0.78
Number of TLS groups	6

**Figure 5:**
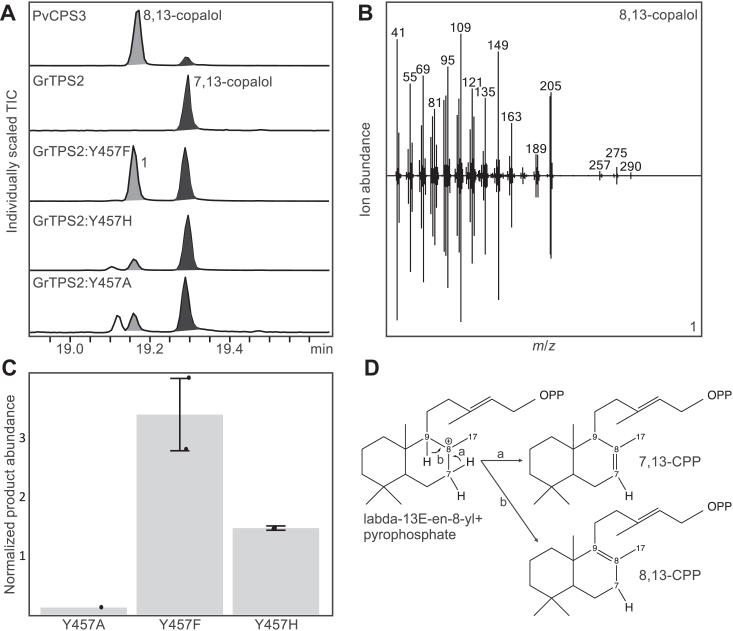
**Functional analysis of GrTPS2 variants of residue Y457.***A*, GC-MS total ion chromatograms (TIC) of 7,13-copalol (*i.e.* dephosphorylated 7,13-CPP, 1), 8,13-copalol (*i.e.* dephosphorylated 8,13-CPP, 2) and an unidentified diterpenoid (3) resulting from *E. coli* co-expression assays of the *Panicum virgatum* 8,13-CPP synthase, PvCPS3 and different variants of GrTPS2 Tyr 457. *B*, mass spectra of 8,13-copalol derived from the activity of PvCPS3 and 8,13-copalol produced by GrTS2:Y457F. *C*, GC-MS quantification of 8,13-copalol produced by GrTPS2 variants Y457F, Y457H, and Y457A as based on normalization against 1-eicosene as internal standard. Error bars represent propagated SE values (n = 3). *D*, predicted mechanism of the conversion of the common landa-13-en-8-yl + to form 7,13-CPP via deprotonation of the endocyclic C-7 methylene or production of 8,13-CP through terminal deprotonation at C-9.

To test the functional impact of these residues, single and select double mutants of GrTPS2 were generated using site-directed mutagenesis to substitute focal residues to either alanine or amino acids present in functionally related class II diTPSs. The generated protein variants were biochemically analyzed using an *Escherichia coli* system engineered for diterpenoid production (36, 37). Specifically, GrTPS2 and the generated protein variants were individually co-expressed for 72 h with 1-deoxy-D-xylulose 5-phosphate reductoisomerase (DXR), 1-deoxy-D-xylulose-5-phosphate synthase (DXS), IPP:DMAPP isomerase (IDI) and the *A. grandis* GGPP synthase to provide enhanced precursor supply (36, 37). Enzyme products were then extracted from the *E. coli* cultures and quantitatively analyzed by GC-MS analysis. The single-residue variants T268H/N/V, F269T/V, T272M, F315H/S/W, and Y457H produced 7,13-CPP at similar or even moderately higher levels as compared to the wild-type GrTPS2 (Fig. 4*B*). By contrast, the substitution of several residues resulted in significantly reduced 7,13-CPP biosynthesis compared to wild-type GrTPS2, including Y209L (38% of wild-type activity), F315A (44%), Y457A (1.5%), Y457F (40%), as well as the double mutant T268F-F269V (5%). Notably, substitution of Y457 to Phe, His or Ala yielded 8,13-CPP as an additional product as based on comparison to an authentic standard enzymatically produced using the 8,13-CPP synthase (PvCPS3) from switchgrass (Fig. 5) (15). The highest formation of 8,13-CPP was detected for variant Y457F, whereas variants Y457H and Y457A produced only small amounts (Fig. 5*C*). In addition, variant Y457A produced small amounts of an additional diterpenoid product, featuring a mass fragmentation pattern most closely matching clerodienyl diphosphate (KPP). However, a comparison to the switchgrass *ent-neo*-KPP synthase, PvCPS1 (15), did not show a product match (Fig. S3). For those protein variants showing substantially reduced activity, additional biochemical and computational tests were performed to assess if the reduced activity is due to the residue exchanges impacting protein expression or structural integrity. Circular dichroism (CD) data were collected for the variants GrTPS2:F315A, GrTPS2:T268F/F269V, GrTPS2:Y209L, GrTPS2:Y457A, and GrTPS2:Y457F to ensure the mutants retain proper folding similarly to wild type GrTPS2. The CD spectrum analysis showed an overlap profile of wild type and protein variants indicating the variants are folded correctly. In addition, the estimated secondary structure using CD data showed the percentage of helix and β-sheets content comparable to the GrTPS2 crystal structure (Fig. S4). In addition, Western Blot analysis of equal volumes of protein expression cultures showed similar protein amounts of GrTPS2 and protein variants (Fig. S4). Furthermore, structural modeling, energy minimization, and calculation of B-values of the above GrTPS2 variants did not indicate any significant structural changes as compared to the crystal structure of wild-type GrTPS2 (Fig. S5). Together, these data indicate that the reduced enzyme activity of these GrTPS2 variants cannot be attributed to a loss of structural integrity or protein expression.

## Discussion

The family of plant class II diTPS enzymes catalyze the protonation-initiated cycloisomerization of the common GGPP precursor to form a range of bicyclic prenyl diphosphate scaffolds that serve as core intermediates in the biosynthesis of thousands of bioactive diterpenoids with functions in general and specialized metabolism (1, 3, 13). Differences in the composition and contour of the class II active site are critical to controlling the coordination of the flexible GGPP substrate to enable precise carbon-carbon rearrangements and yield scaffolds of distinct structure and stereochemistry (13). Structural knowledge of *G. robusta* 7,13-CPP synthase, GrTPS2, expands our understanding of the functional divergence of the plant diTPS family and provides resources for protein and pathway engineering for bioactive natural products. The crystal structure of GrTPS2 adds a third protein structure of diTPSs that feature a functional class II active site, including the previously reported structures of the class II *ent*-CPP synthase, AtCPS, and the bifunctional class II/I enzyme, *A. grandis* abietadiene synthase (17, 18, 19). Significant similarity of the overall structure of GrTPS2 at 2.1Å of resolution as compared to AtCPS (RMSD 1.95Å at 45% protein sequence identity) highlights the conserved fold of class II diTPSs adopting a common α-helical γβα-domain architecture with the active site situated at the γ- and β-domain (Fig. 2). The presence of a solvent channel in GrTPS2 (Fig. 3) is shown to connect the catalytic DIDD motif with bulk solvent similar to AtCPS. The conservation of the residues involved in the solvent channel coordination suggests that the mechanism by which the general acid Asp residue of the DIDD motif is reprotonated during catalysis is conserved in GrTPS2. The lack of two water molecules contributing to this solvent channel may be due to the higher 2.1Å of resolution as compared to the 1.55Å structure of AtCPS (19).

Site-directed mutagenesis of select residues provided insight into the amino acid environment of the GrTPS2 active site that determines overall catalysis and stereospecific formation of 7,13-CPP (Figs. 4 and 5). Among these Y209 and T268 are located in place of the HN dyad shown to control stereospecificity in *ent*-CPP synthases (25, 30, 32). Although no impact on GrTPS2 product specificity was observed when exchanging Y209 for a smaller Leu residue present in known 8,13-CPP synthases (Fig. S2), a significant decrease in 7,13-CPP formation demonstrates a catalytic role of Y209. By contrast, only a mutation of T268 for a Phe residue (as detected in a T268F/F269V double mutant) caused a substantial activity decrease, suggesting that the amino acid side chain is not critical in this position, except for bulkier side chains that appear to interfere with catalysis. Exchange of the neighboring F269 did not alter GrTPS2 activity or product specificity, differing from class II diTPSs from several Lamiaceae species, where Thr residues in this position impact sterecontrol (32), as well as *A. grandis* abietadiene synthase, where mutation of H348 in this position causes water quenching of the intermediary labda-13-en-8-yl^+^ carbocation rather than terminal deprotonation at C-17 to form (+)-CPP (28). In addition, decreased 7,13-CPP formation when replacing F315 with Ala but not Ser, His or Trp residues indicate a contribution but not critical role of this active site position in GrTPS2 function. An impact of F315 on product specificity, as shown for the respective F331 in the switchgrass 8,13-CPP synthase, PvCPS1, and F357 in the KPP synthase, PvCPS1 (15), was not observed. In addition, the exchange of Y457 to His, Phe and Ala caused not only a decrease in 7,13-CPP formation but also a shift to the formation of the 8,13-CPP isomer (Fig. 5). While the removal of a larger side chain through Ala substitution resulted in a nearly complete loss of function, exchange to His or Phe showed activity loss to a lesser extent and increased production of 8,13-CPP, suggesting that the hydroxyl group of Y457 and presence of an aromatic ring in this position make an important contribution to directing catalysis toward deprotonation of labda-13-en-8-yl^+^ at the endocyclic C-7 methylene to form 7,13-CPP. This conclusion is supported by the previous mutagenesis studies that showed the respective His476 residue in this position to contribute to altering product specificity of the 8,13-CPP synthase, PvCPS3, toward 7,13-CPP production (15). Additionally, this active site position is also occupied by a His residue (His501) in the rice *syn*-CPP synthase, OsCPS4, where mutagenesis of this position demonstrated a critical function in defining stereocontrol during catalysis (29).

The crystal structure of GrTPS2, producing the rare 7,13-CPP intermediate en route to grindelic acid as the signature metabolite of species of *Grindelia*, expands our understanding of the catalytic mechanism and functional divergence of the family of class II diTPSs involved in general and specialized diterpenoid biosynthesis. Identification of active site residues that may control overall catalytic activity and product specificity of GrTPS2 provides insights into the active site composition that distinguishes GrTPS2 from other class II diTPSs and direct catalysis toward the production of 7,13-CPP.

## Experimental procedures

### GrTPS2 expression and purification

GrTPS2 (Genbank: KR089902) with the native, full-length gene sequence was inserted into the pSKB3 plasmid and expressed using LB autoinduction media in *E. coli* Bl21DE3 or Rosetta2 DE3 cells for 24 h at 15 °C in 2 L baffled flasks. Cells were pelleted and stored at −80 °C. For purification, cells were thawed and re-suspended by stirring with a stir bar in 25 mM Tris, pH 8.0, 250 mM NaCl, 2 mM MgCl_2_, 1 mM DTT, and 1× Novagen protease inhibitors cocktail, 0.1 mg/ml lysozyme, 3 μg/ml DNase I. Cells were lysed by multiple passes in the Emulsiflex C3 emulsifier. The lysate was clarified by centrifugation at 40,000*g* for 40 min. After adding 20 mM imidazole, the clarified lysate was loaded onto a 5 ml Histrap column on an AKTA purifier FPLC instrument. The Histrap column was equilibrated with base buffer prior to loading of the lysate. The bound protein was eluted with a 0 to 50%B gradient in 20 CV. Buffer B was the same as a base buffer with the addition of 0.5 M imidazole. The elution peak was analyzed by SDS-PAGE and the cleanest fractions were pooled prior to dialysis against 900 ml of 25 mM Tris, pH 8.0, 25mM NaCl, 2 mM MgCl_2_, 1 mM DTT for two 1-h periods at 4 °C. The dialyzed protein was 0.45 μM filtered to remove any aggregates prior to loading onto a 5 ml Q ion exchange column on the AKTA purifier FPLC instrument. The bound protein was eluted with a 0 to 45% B in 24 CV. Buffer B was the same as the dialysis buffer but NaCl was 1 M. The elution fractions were analyzed by SDS-PAGE and the cleanest fractions were pooled, concentrated, and injected onto a 10 × 300 Superdex300 size exclusion column (SEC). The column was pre-equilibrated with a base buffer prior to injecting the protein. The SEC column was run at 0.5 ml/min and the injection volumes were ∼1 ml. The cleanest fractions as determined by SDS-PAGE analysis were pooled and the His-tag was cleaved off by the tobacco etch virus (TEV) protease. The cleaved material was 0.45 μM filtered prior to passing through a 1 ml Histrap column on the bench to remove the uncleaved protein as well as the His-tagged TEV protease. The GrTPS2 protein with the His-tag removed was concentrated with a 50 kD centrifugal concentrator to 14 mg/ml. This material was used for crystallization trials.

### Crystallization, X-Ray data collection, and structure determination

The purified GrTPS2 protein was screened against the crystallization set of solutions: Berkeley Screen (38), MCSG-1 (Anatrace), ShotGun (Molecular Dimensions), Crystal Screen, PEG/Ion, Index, and PEGRx (Hampton Research). Crystals of GrTPS2 were found in 0.2 M sodium acetate, 0.1 M Tris pH 8.5, 30% PEG 4000. The crystal of GrTPS2 was placed in a reservoir solution containing 20% (v/v) glycerol, then flash-cooled in liquid nitrogen. The X-ray data set for GrTPS2 was collected at the Berkeley Center for Structural Biology beamline 8.2.1 at the Advanced Light Source at Lawrence Berkeley National Laboratory. The diffraction data were processed using the program Xia2 (39). The crystal structure of GrTPS2 was solved by molecular replacement with the program PHASER (40, 41) using initial coordinates from the PDB ID 3PYA. The atomic positions obtained from the molecular replacement were used to initiate refinement using the Phenix suite (40). Structure refinement was performed using the phenix.refine program (40, 42). Manual rebuilding was done using COOT (43). Root-mean-square deviations from ideal geometries for bond lengths, bond angles, and dihedral angles were calculated with Phenix (42). The stereochemical quality of the final model of GrTPS2 was assessed by the program MOLPROBITY (44). A summary of crystal parameters, data collection, and refinement statistics can be found in Table 1.

### GrTPS2 ligand docking and site-directed mutagenesis

Ligand docking of the substrate analog, 15-aza-14,15-dihydro-geranylgeranyl thiolodiphosphate, within the class II active site of GrTPS2 was performed using Molegro Virtual Docker with standard docking parameters and a manually positioned 12Å search space around the active site (45). GrTPS2 protein variants were generated by gene synthesis or, where required, whole-plasmid PCR amplification with site-specific sense and antisense oligonucleotides (see below) using the NEB Q5 Site-Directed Mutagenesis Kit (New England Biolabs) and an N–terminally truncated version of GrTPS2 (lacking the plastidial signal peptide; Δ47) in the pET28b(+) expression vector (EMD Millipore) (9). Treatment with *Dpn*I was used to remove template plasmids. All generated protein variants were sequence-verified (Quintara Biosciences, South San Francisco, CA) before functional analysis. Plasmids were prepared using the Monarch Plasmid Miniprep kit (New England Biolabs) and transformed into chemically competent *E. coli* Bl21DE3 C41 cells. Oligonucleotides used for site-directed mutagenesis included: GrTPS2:F315H (For: AGGAATCTCGCATGCTAGAAAATG; Rev: TTTTGATCCCAATATCTGTAG) and GrTPS2:T268F/F269V (T268F, For: AGGAGTTCCATTTTTTTACCCAACTG, Rev: CCATTAAATTTGGCAACAAG; F269V, For: AGTTCCATTTGTGTACCCAACTG, Rev: CCTCCATTAAATTTGGCAAC).

### Enzyme co-expression assays

Co-expression of GrTPS2 protein variants was conducted in an *E. coli* platform engineered for diterpenoid production (36, 37). Each GrTPS2 variant was co-expressed with the pIRS plasmid (carrying key genes of the MEP pathway, namely 1-deoxy-D-xylulose 5-phosphate reductoisomerase (DXR), 1-deoxy-D-xylulose-5-phosphate synthase (DXS), and IPP:DMAPP isomerase (IDI)) and pACYCDUET-pGG encoding for a *A. grandis* GGPP synthase as described elsewhere (36, 37). Constructs were co-transformed into *E. coli* Bl21DE3 C41 cells and cultures were grown at 37 °C and 180 rpm in 50 ml Terrific Broth (TB) media, pH 7.5 until an OD_600_ of ∼0.5 to 0.6 before cooling to 16 °C. Cultures were incubated at 16 °C for 45 min before induction with 1 mM isopropyl-β-D-1-thiogalacto-pyranoside (IPTG) and 1 mM MgCl_2_. After 72 h of incubation, enzyme products were extracted using hexane with 0.35 ng/μl 1-eicosene as internal standard and air-dried for GC-MS analysis. For analysis of protein expression levels, equal culture volumes from the wild-type GrTPS2 and protein variants were taken after 24 h post-induction and cells were harvested via centrifugation at 8000*g* at 4 °C for 15 min. After lysis through sonication, cell lysates were collected by centrifugation at 16,000*g* at 4 °C for 15 min and separated by SDS-PAGE. Western Blot analysis was then performed using a Trans Blot Turbo System (Biorad) using a monoclonal mouse anti-His-alkaline phosphatase antibody.

### GC-MS analysis

Extracted enzyme products were analyzed on an Agilent 7890B GC interfaced with a 5977 Extractor XL MS Detector at 70 eV and 1.2 ml/min He flow, using an Agilent DB-XLB column (30 m, 250 μm i.d., 0.25 μm film) and the following GC parameters: 50 °C for 3 min, 15 °C/min to 300 °C, hold 3 min with pulsed splitless injection at 250 °C. MS data from 40 to 400 mass-to-charge ratio (*m*/*z*) were collected after a 13 min solvent delay. Enzyme products, specifically 8,13-copalol, 7,13-copalol, were identified based on matching mass spectra and retention times as compared to enzymatically produced authentic standards. Notably, the initial 8,13-CPP and 7,13-CPP diTPS products are typically dephosphorylated by endogenous *E. coli* phosphatases during the 72-h incubation, enabling the direct organic solvent extraction and GC-MS analysis of the resulting diterpene alcohols without the need for additional modifications.

### Circular dichroism (CD) spectroscopy

Rosetta2 DE3 cells transformed with the plasmids coding for the wild type and mutant GrTPS2 genes were grown at 37 °C in 2L baffled flasks containing 1L Terrific Broth with kanamycin and chloramphenicol. The cultures were induced with 0.3 mM IPTG when the OD_600_ was ∼1.0. The flasks were moved to an 18 °C incubator and expression continued for about 20 h. Enzymes used for the CD studies were purified by nickel affinity followed by ion exchange chromatography on the AKTA PURE system. For CD spectra collection and analysis, the purified enzymes were diluted to 0.11 mg/ml, or 1.2 μM in 5 mM Tris, pH 8.0, 10 mM NaCl. The protein solution was placed in a 1.0 mM path length cuvette and the CD spectrum was collected from 260 – 190 nM at 15 °C in a Jasco J-815 CD Spectrometer. Scanning speed was 100 nM/min with 1 nM bandwidth. Data that was buffer corrected and smoothed was analyzed by BeStSel server (46).

### Protein modeling and minimization

Using the wild-type GrTPS2 crystal structure as a basis, the structures of selected single mutants (GrTPS2:Y269L, GrTPS2:F315A, GrTPS2:Y457A, GrTPS2:Y457F) and the double mutant, GrTPS2:T268F/F269V) were generated in PyMOL. These mutants were then minimized using the Yasara1 software package. The minimized mutant structures were superimposed with the wild-type GrTPS2/aza-GGSPP docked complex. All structure analyses and figures were created using PyMOL v 3.0 (47). A summary of the potential energy values of the minimized mutants can be found in Fig. S5.

### Sequence alignments

Amino acid sequence alignments were performed using Geneious 2023.1.2 (https://www.geneious.com) with default parameters. Sequence alignments were visualized using the seqvisr package (https://zenodo.org/doi/10.5281/zenodo.6583980) in R Statistical Software 2023.09.0  + 463 (https://github.com/vragh/seqvisr).

## Data availability

Coordinates and structure factors of GrTPS2 can be found in the Protein Data Bank under accession number 9B99.

## Supporting information

This article contains supporting information.

## Conflict of interest

The authors declare that they have no conflicts of interest with the contents of this article.
