# Supplementary material for: The crystal structure of Grindelia robusta 7,13-copalyl diphosphate synthase reveals active site features controlling catalytic specificity
Source: J Biol Chem. 2024 Oct 23;300(12):107921. doi: 10.1016/j.jbc.2024.107921 (PMC11599460; doi:10.1016/j.jbc.2024.107921)
Supplement: Supporting information [file mmc1.docx]

**Supporting Information**

**The crystal structure of *Grindelia robusta* 7,13-copalyl diphosphate synthase reveals active site features controlling catalytic specificity.**

**Anna E. Cowie^1#^, Jose H. Pereira^2,3#^, Andy DeGiovanni^2,3^, Ryan P. McAndrew^2^, Malathy Palayam^1^, Jedidiah O. Peek^1^, Andrew J. Muchlinski^1^, Yasuo Yoshikuni^4^, Nitzan Shabek^1^, Paul D. Adams^2C,3,5^, Philipp Zerbe^1^***


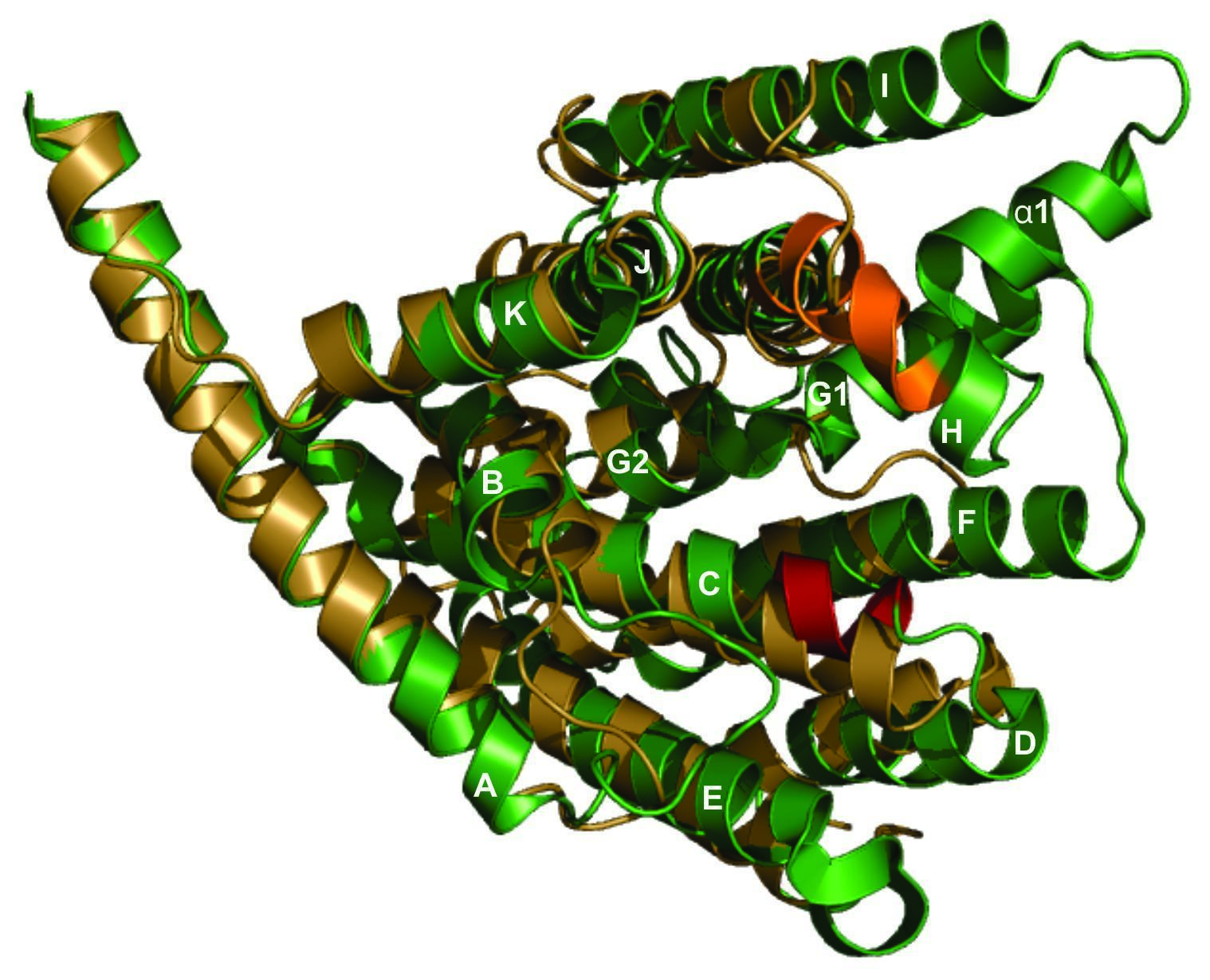


**Supporting Fig. S1.** Structural comparison of the GrTPS2 ɑ-domain. Superposition of the ɑ-domain structures of GrTPS2 (brown) and the bifunctional class II/I diTPS *Abies grandis* abietadiene synthase (purple; PDB ID 3S9V). The class I active site DDxxD (magenta) and NSE/DTE (orange) motifs absent in GrTPS2 are highlighted.

**
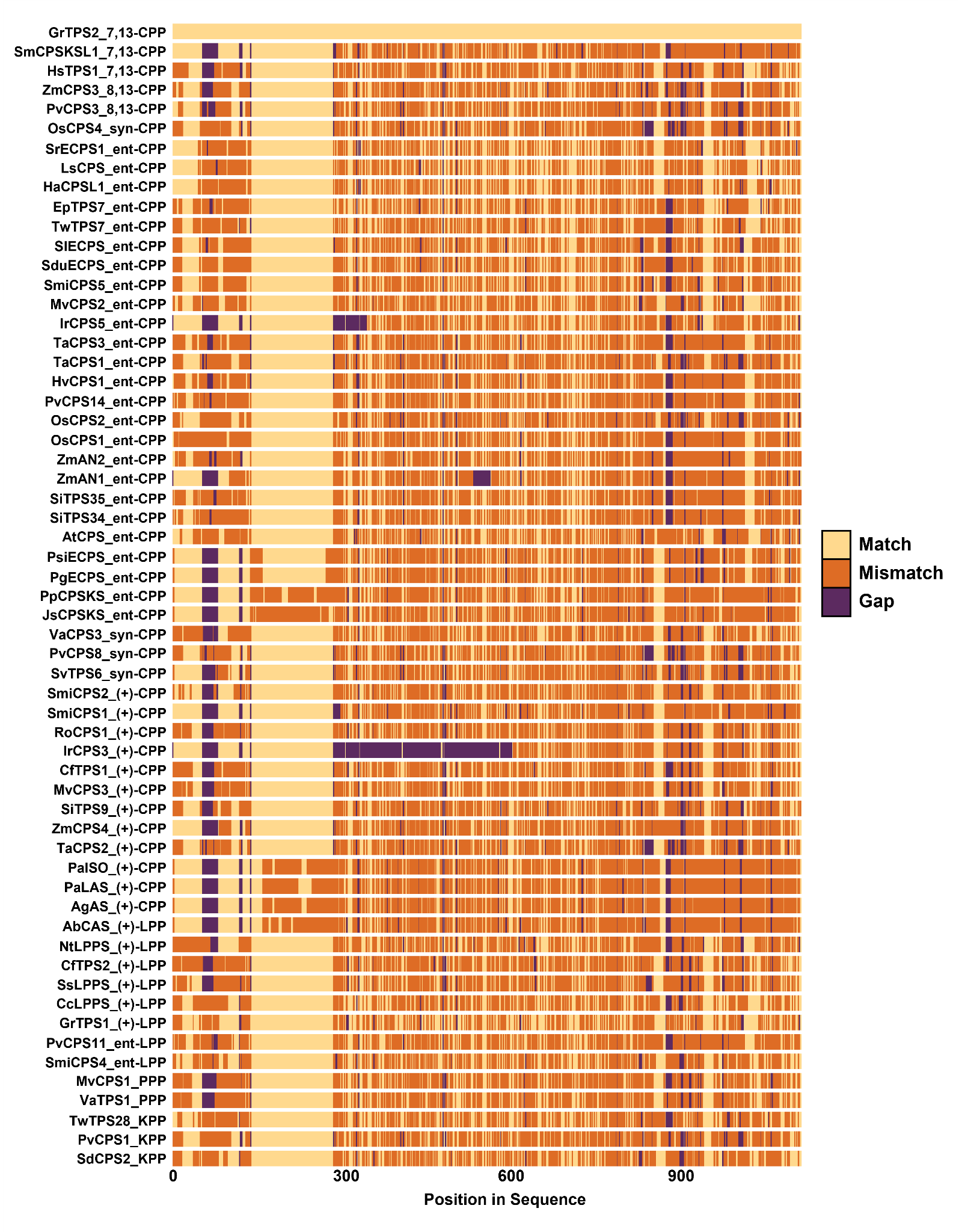
**

**Supporting Fig. S2.** Amino acid sequence alignment of GrTPS2 and known plant class II diterpene synthases. CPP, copalyl diphosphate; CPS, copalyl diphosphate synthase; KSL, kaurene synthase-like. Species abbreviations: Gr, *Grindelia robusta*; Hs, *Hyptis suaveolens*; Sm, *Selaginella moellendorffii*; Pv, *Panicum virgatum*; Zm, *Zea mays*; Js, *Jungermannia* *subulata*, Pp, *Physcomitrella* *patens*; Pg, *Picea* *glauca*; Psi, *Picea* *sitchensis*; At, *Arabidopsis* *thaliana*;, Si, *Setaria* *italica*; Os, *Oryza* *sativa*; Hv, *Hordeum* *vulgare*; Ta, *Triticum* *aestivum*; Ir, *Isodon* *rubescens*; Mv, *Marrubium* *vulgare*, Smi, *Salvia* *miltiorrhiza*; Sdu, *Scoparia* *dulcis*; Sl, *Solanum* *lycopersicum*; Tw, *Tripterygium* *wilfordii*; Ep, *Euphorbia* *peplus*; Ha, *Helianthus* *annuus*; Ls; *Lactuca* *sativa*; Sr, *Stevia* *rebaudiana*; Sv, *Setaria* *viridis*; Va, *Vitex* *agnus-castus*; Ag, *Abies* *grandis*, Pa, *Picea* *abies*; Cf, *Coleus* *forskohlii*; Ro, *Rosmarinus* *officinalis*; Sd, *Salvia* *divinorum*; Cc, *Cistus* *creticus*; Nt, *Nicotiana* *tabaccum*; Ab, *Abies* *balsamea*.

**
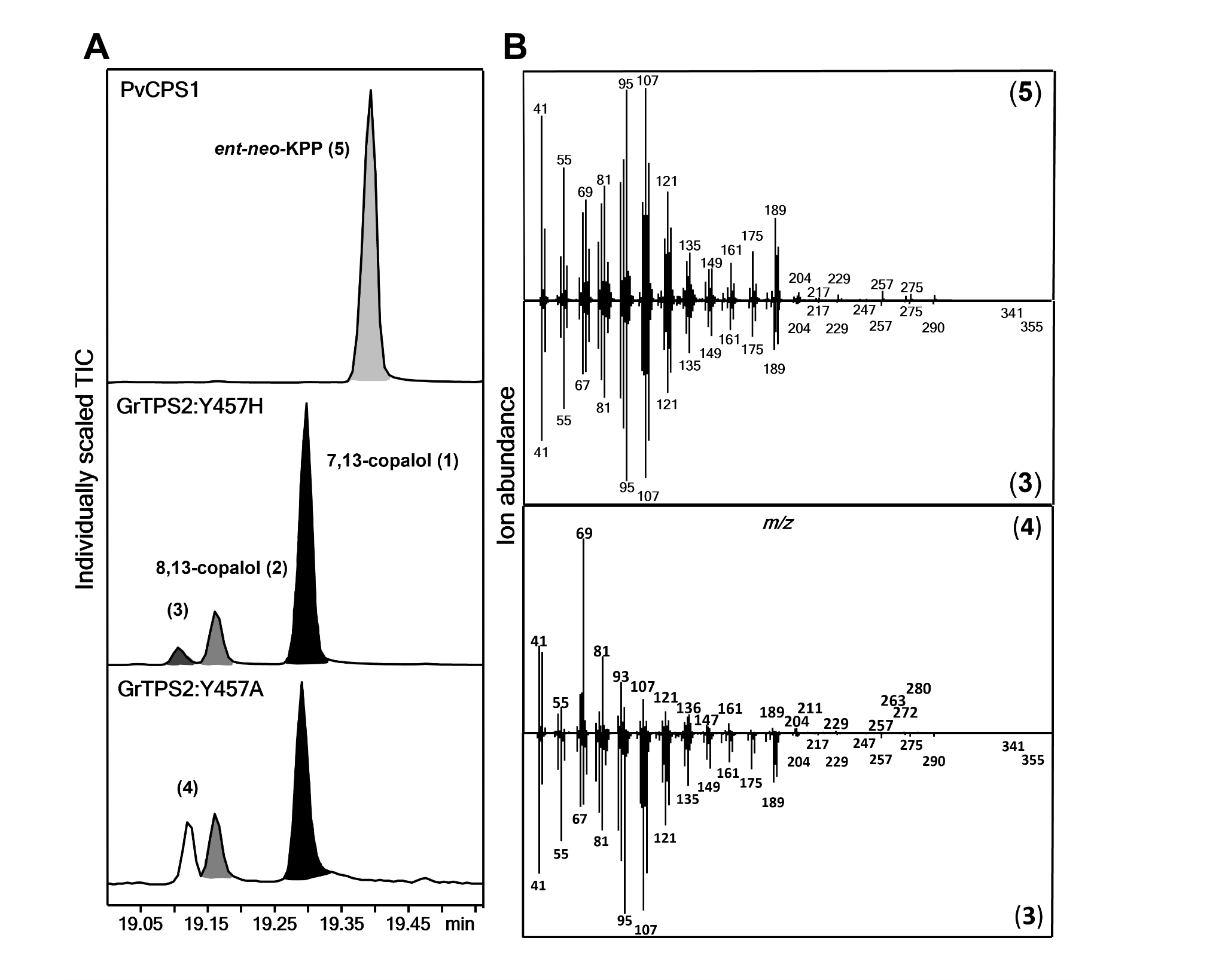
**

**Supporting Fig. S3.** Biochemical characterization of GrTPS2 protein variants Y457H and Y457A as compared to the switchgrass *ent-neo*-KPP synthase, PvCPS1. (**A**) GC-MS total ion chromatograms (TIC) of 7,13-copalol (i.e. dephosphorylated 7,13-CPP [**1]**), 8,13-copalol (i.e. dephosphorylated 8,13-CPP, [**2]**), an unidentified diterpenoid [**3**] resulting from *E. coli* co-expression assays of the switchgrass 8,13-CPP synthase, PvCPS3, and different variants of GrTPS2 Y457, and a unidentified predicted prenyldiphosphate-derivative [**4**]. (**B**) Mass spectrum products (**3 & 4**).

**A**


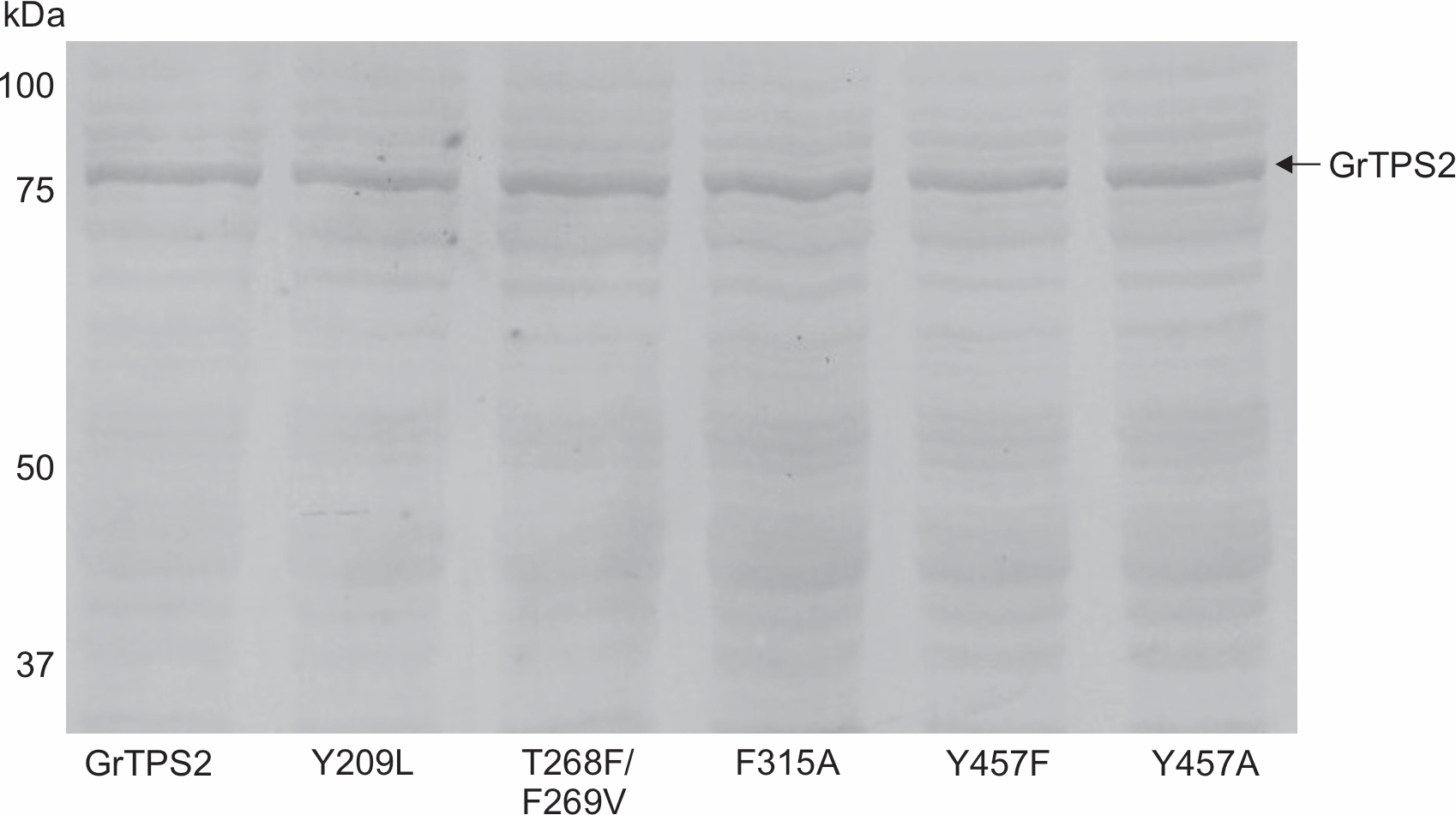


**B**

**C**

|  | **GrTPS2 crystal structure** | **Wild Type** | **F315A** | **T268N/ F269V** | **Y209L** | **Y457F** | **Y457A** |
| --- | --- | --- | --- | --- | --- | --- | --- |
| Helix | 61.2 | 63.2 | 60.5 | 58.9 | 63.7 | 66.2 | 65 |
| β-sheet | 0 | 0 | 0 | 0 | 0 | 0 | 0 |

**Supporting Fig. S4.** Structural analysis of wild type GrTPS2 and select protein variants. (**A**) Western blot analysis of affinity-purified GrTPS2 and protein variants using a monoclonal α-(His)_5_-antibody. (**B**) Circular dichroism (CD) spectra for single amino acid substitution variants as compared to wild type GrTPS2. (**C**) Estimated secondary structure content (%) using CD data for the wild type, protein variants and the GrTPS2 crystal structure.

**Supporting Fig. S5.** Structural analysis of wild type GrTPS2 with predicted models of GrTPS2 protein variants. (**A**) Crystal structure of GrTPS2 (light purple) with the docked substrate analog *aza*-GGSPP (light orange). (**B**) Superposition of wild type GrTPS2 (light purple) with the predicted structures of variants (i) GrTPS2:Y209L (green), (ii) GrTPS2:F315A (magenta), (iii) GrTPS2:T362M (brown), (iv) GrTPS2:A453G (light yellow), (v) GrTPS2:Y457A (cyan), (vi) GrTPS2:Y457F (teal), and (vii) GrTPS2:T268F/F269V (light pink). (**C**) Minimized protein variant structures of GrTPS2. Energy minimization was performed in Autodock Vina and structural analysis was conducted using PyMoL version 3.0
